# The distinct transcriptome of virulence-associated phylogenetic group B2 *Escherichia coli*

**DOI:** 10.1128/spectrum.02085-23

**Published:** 2023-09-19

**Authors:** Jacob Hogins, Zhenyu Xuan, Philippe E. Zimmern, Larry Reitzer

**Affiliations:** 1 Department of Biological Sciences, The University of Texas at Dallas, Richardson, Texas, USA; 2 Department of Urology, The University of Texas Southwestern, Dallas, Texas, USA; Forschungszentrum Jülich GmbH, Jülich, Germany

**Keywords:** *Escherichia coli*, pathogens, urinary tract infection, transcriptional regulation, transcription factors, phylogenetic analysis, metabolism

## Abstract

*Escherichia coli* strains of phylogenetic group B2 are often associated with urinary tract infections (UTIs) and several other diseases. Recent genomic and transcriptomic analyses have not suggested or identified specific genes required for virulence, but have instead suggested multiple virulence strategies and complex host-pathogen interactions. Previous analyses have not compared core gene expression between phylogenetic groups or between pathogens and nonpathogens within phylogenetic groups. We compared the core gene expression of 35 strains from three phylogenetic groups that included both pathogens and nonpathogens after growth in a medium that allowed comparable growth of both types of strains. K-means clustering suggested a B2 cluster with 17 group B2 strains and two group A strains; an AD cluster with six group A strains, five group D strains and one B2 strain; and four outliers which included the highly studied model uropathogenic *E. coli* strains UTI89 and CFT073. Half of the core genes were differentially expressed between B2 and AD cluster strains, including transcripts of genes for all aspects of macromolecular synthesis—replication, transcription, translation, and peptidoglycan synthesis—energy metabolism, and environmental-sensing transcriptional regulators. Notably, core gene expression between nonpathogenic and uropathogenic transcriptomes within phylogenetic groups did not differ. If differences between pathogens and nonpathogens exist, then the differences do not require transcriptional reprogramming. In summary, B2 cluster strains have a distinct transcription pattern that involves hundreds of genes. We propose that this transcription pattern is one factor that contributes to virulence.

**IMPORTANCE:**

*Escherichia coli* is a diverse species and an opportunistic pathogen that is associated with various diseases, such as urinary tract infections. When examined, phylogenetic group B2 strains are more often associated with these diseases, but the specific properties that contribute to their virulence are not known. From a comparative transcriptomic analysis, we found that group B2 strains grown in a nutrient-rich medium had a distinct transcription pattern, which is the first evidence that core gene expression differs between phylogenetic groups. Understanding the consequences of group B2 transcription pattern will provide important information on basic *E. coli* biology, the basis for *E. coli* virulence, and possibly for developing therapies for a majority of urinary tract infections and other group B2-associated diseases.

## INTRODUCTION

Urinary tract infections (UTIs) are common bacterial infections that often recur (1). A UTI requires rapid bacterial growth in the bladder; extensive interactions with urothelial cells which include attachment, entry, survival, and rapid intracellular replication; and release back into the bladder (2). Only one in 10^5^–10^6^ cells survive passage through urothelial cells, and the mechanism of intracellular survival is poorly understood. Antibiotic treatment usually resolves UTIs but also increases the risk of bacteria developing multidrug resistance (3, 4). Recurrent UTIs (RUTIs) can last for years and become hard to control, in part, because of antibiotic recalcitrance (5). The WHO recognizes UTIs as loci for development of multidrug-resistant bacteria which can impact the outcome of other clinically important diseases that are treated with the same antibiotics (6). Alternate or adjunct therapies are urgently needed (2, 3, 7), but potential targets of therapy have been elusive, in part because of an incomplete understanding of virulence.

*Escherichia coli*, which causes most UTIs and RUTIs (3, 8), is a diverse species that can be divided into phylogenetic groups (9, 10). Although strains from several phylogenetic groups are pathogenic (11, 12), group B2 strains are more prevalent in UTIs and RUTIs (8, 11, 12), as well as in *E. coli*-caused neonatal meningitis (13, 14), inflammatory bowel diseases (Crohn’s disease and ulcerative colitis) (15 – 17), and eye infections (18), are more often associated with colorectal cancer (19), and have more characteristics associated with multiple antibiotic resistance and extended spectrum β-lactamase production (20). Paradoxically, a phylogenetic evolutionary analysis found that group B2 strains have lost several environmental-adaptive genes, including genes for survival in extreme conditions of low pH, high temperature, and low osmolarity (21). This phylogenetic study proposed that different lineages had distinct adaptive trajectories, but provided no explanation for the advantage of the group B2 adaptive strategy.

A common strategy to understand virulence is to identify genes that are only present in pathogens, which assumes that acquisition of certain genes will endow nonpathogenic strains with pathogenic potential. This type of virulence gene is by definition an accessory gene, i.e., not present in all *E. coli* strains. However, virulence factor genes for uropathogenic *E. coli* (UPEC) have been frustratingly difficult to identify. More than two decades of searches for virulence genes culminated in a study that concluded that UPEC strains do not have a common set of virulence genes and that previous analyses could not distinguish virulence genes from group B2-specific accessory genes (22).

An alternate or, more accurately, a complementary hypothesis is that UPEC strains have a common set of more highly expressed genes. If virulence is a property of strains from different phylogenetic genetic groups, then this hypothesis assumes cores genes are more highly expressed in pathogenic strains. The first test of this hypothesis did not identify genes from urine-associated *E. coli* whose expression correlated with quantifiable properties of murine infections (22). The authors then proposed multiple virulence strategies based on a complex interplay between hosts and UPEC strains (22). This conclusion could serve as a definition of virulence for an opportunistic pathogen. A second transcriptomic analysis postulated that expressed virulence genes would only be apparent from bacteria isolated directly from the human host. UPEC RNA from symptomatic patients was extracted immediately after urine collection (*in vivo*) and compared to RNA extracted after growth in pooled urine (*ex vivo*) and a rich nutrient broth. The results were interpreted to suggest a transcriptional program for UPEC strains which had upregulated genes for rapid growth, such as genes for the translational machinery (23). Rapid growth has been proposed to be a virulence factor, but slow-growing strains have also been associated with UTIs (24 – 26), which suggests that rapid growth may not be sufficient for UTIs.

Our primary goal was to determine whether core gene expression differs between strains from group B2 and other groups because of the association of group B2 strains with a variety of diseases. Our secondary goal was to test the hypothesis that intracellular survival during an infection selects for strains that differ from their nonpathogenic relatives, and to this end, we compared core gene transcriptomes from UPEC and nonpathogenic *E. coli* (NPEC) strains within phylogenetic groups. Our primary conclusions are that group B2 strains generally had a distinct transcriptomic pattern, and that UPEC and NPEC transcriptomes were generally indistinguishable. In other words, our results show that B2 strains have a distinct transcription program which we propose results in a greater virulence potential than strains from other groups.

## RESULTS

### Comparisons between and within phylogenetic groups

We compared the transcriptomes from 35 strains: 14 NPEC and 9 UPEC strains from the ECOR collection, nine recently isolated strains causing RUTIs, the well-studied model UPEC strains CFT073 and UTI89, and the nonpathogenic lab strain W3110. Groups A, B2, and D were represented by 9, 20, and 6 strains, respectively. We employed ECOR strains because (i) they are readily available to the research community, and (ii) they contain UPEC and NPEC strains from different phylogenetic groups.

We assumed that major differences of core gene expression would be readily detectable regardless of the growth medium. Because previous studies have shown that UPEC strains grow faster in urine and other media (27 – 29), an important consideration for selecting a growth medium was for comparable growth rates for NPEC and UPEC strains. The medium was 0.5% glucose-1% tryptone-0.25% NaCl, and with few exceptions, all strains grew at the same rate. We grew cultures aerobically because direct measurement suggests 4%–7% oxygen content in the bladder, and because aerobic growth is easier to reproduce between labs (30, 31).

**Fig 1:**
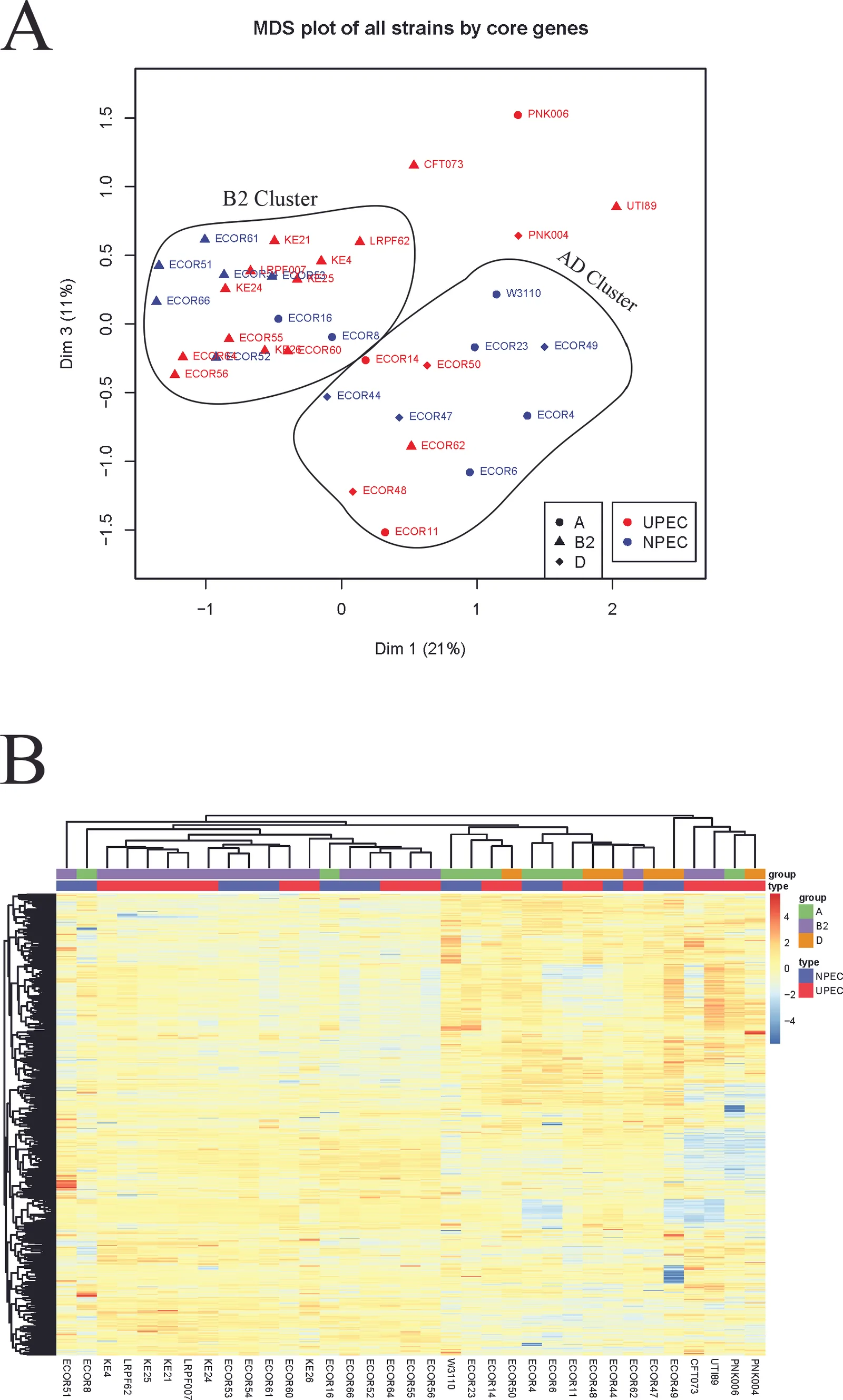
Visualizations of the total transcriptomes of 35 strains of *E. coli*. (**A**) Dimensional reductive analysis visualized by a MDS plot of these transcriptomes identifies two transcriptomic clusters labeled here as the B2 and AD clusters and four strains that do not form a separate cluster as determined by K-mean analysis. (**B**) Visualization of the top 1,000 most variable genes identified three branches corresponding to the clusters previously described. Two strains (ECOR 8 and 16) are genomically group A, but transcriptomically cluster B2, and one strain (ECOR62) is genomically group B2, but transcriptomically cluster AD.

Comparison across phylogenetic groups requires analysis of shared genes, i.e., core genes. Two thousand six hundred sixty-seven genes were present in at least 34 of the 35 strains and were defined as core genes (Table S1). A multidimensional scaling (MDS) plot of core gene expression shows a B2 cluster with 17 B2 strains and two group A NPEC strains, and an AD cluster with 6, 5, and 1 strains from groups A, D, and B2, respectively (Fig. 1A). We confirmed the phylotypes of the two group A strains in the B2 cluster and the B2 strain in the AD cluster. Four UPEC strains—CFT073, PNK004, PNK006, and UTI89—mapped outside of the B2 and AD clusters, but K-mean clustering did not define these strains as a group. A heatmap of the 1,000 most variably expressed genes visually shows the differences between the AD and B2 clusters (Fig. 1B). Patterns of gene expression were similar between ECOR and recently isolated UPEC strains, which suggests that the ECOR strains have not undergone extensive lab evolution since their isolation. We conclude that (i) transcriptomic and phylogenetic groupings are similar, but not identical, and (ii) most B2 strains not only differ from most group A and D strains in accessory genes, which define the phylogenetic groups, but also in the pattern of core gene expression.

One thousand three hundred forty (50.2%) of core genes were differentially expressed genes (DEGs) [false discovery rate (FDR) <0.05] between the B2 and AD cluster strains (Table S1, DEG tab). Gene ontology analysis indicated that B2 cluster strains had higher expression of genes for translation (e.g., ribosome protein genes), several biosynthetic pathways (purines, glutamine, glycine, and ribose), iron acquisition, carbohydrate transport, and transport processes that involve protons, but lower expression of several genes for environmentally responsive transcription factors (TFs) (Table S1, GOMF and GOBP tabs).

Differences in core gene expression did not distinguish UPEC from NPEC strains. Within the B2 cluster, 61 genes with known functions (excluding y genes whose products have no known function) were differentially expressed (FDR < 0.05) between UPEC and NPEC strains (Table S2). Forty-two of these genes code for components of the flagella and chemotaxis machinery. The expression of motility genes is anomalous because the growth medium contained glucose which should prevent cyclic-AMP synthesis and flagella expression. Only 3 of the 19 B2 cluster strains expressed motility genes, and when these strains were removed from the analysis, UPEC and NPEC strains did not differentially express these 42 genes. Similarly, 10 of the 61 genes code for cyclic-AMP-regulated catabolic enzymes, and in this case, one strain (ECOR51) greatly distorted the statistics. When ECOR51 results were excluded, these 10 genes were not only not differentially expressed, but they were not expressed. The remaining nine genes were expressed in the recently isolated UPEC strains, but not in the ECOR UPEC strains. These genes are worthy of further analysis but have not been previously associated with virulence. We conclude that identification of a DEG not only requires a statistical analysis, but also an examination of transcripts from individual strains to ensure against false positives. For this reason, when we identify DEGs, we show the transcript levels for genes in individual B2 and AD cluster strains. Within the AD cluster, no genes were differentially expressed between UPEC and NPEC strains (Table S3).

The transcriptomes of four outlier UPEC strains—CFT073, PNK004, PNK006, and UTI89—are from three different phylogenetic groups. A major difference between CFT073 and other B2 strains was 47-fold more *phoB* transcripts. PNK004’s transcriptome is distorted by extraordinarily high expression of *rcsA*, which codes for a regulator of capsule synthesis, and genes of colanic acid. UTI89 and PNK006 did not have major expression distortions that were easily identified. In any case, these strains are not representative of most UPEC strains.

In summary, the transcriptomes of most strains clustered into two major groups which generally, but not always, followed the phylogenetic grouping. No obvious differences in core gene expression existed between UPEC and NPEC strains of the same phylogenetic group. The remaining sections describe the expression differences between B2 and AD cluster strains with a focus on the genes of macromolecular synthesis, macromolecular structure, and energy metabolism.

### Macromolecular synthesis and structure

#### Translation

**Fig 2:**
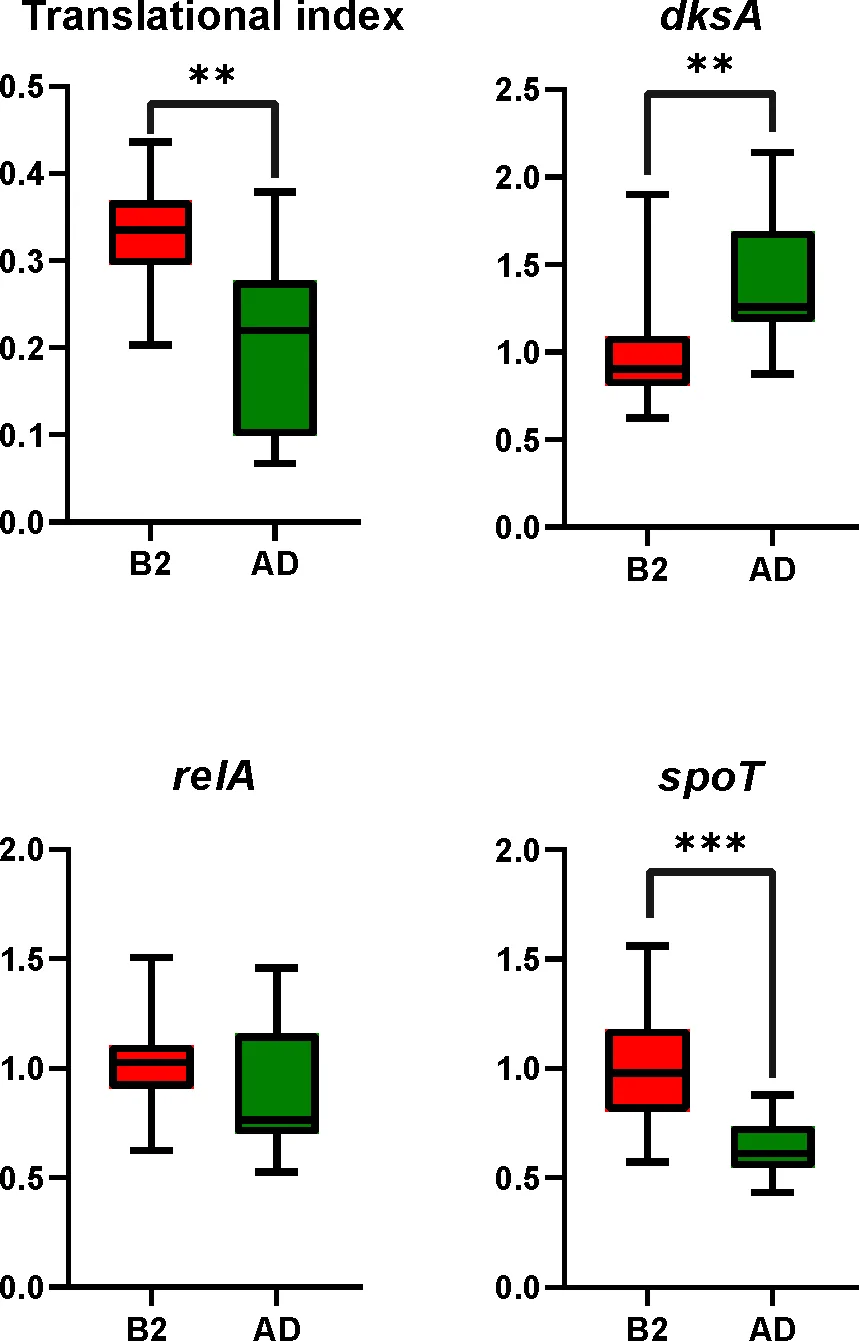
Comparison of transcripts from genes for ribosomal proteins and *dksA*, *relA*, and *spoT*. The translational index is the normalized sum of transcripts for proteins of the ribosome small and large subunits. For *dksA*, *relA*, and *spoT*, the transcripts per million counts are normalized to the average transcripts for B2 cluster strains. *P < 0.05; **P < 0.01; ***P < 0.001. Statistical significance was determined by Mann-Whitney test because we assumed a non-Gaussian distribution and unequal variance. Transcript values for all B2 and AD cluster strains were used with no exclusions.

Gene ontology indicated that B2 cluster strains had greater expression of genes for the translational machinery and nucleotide precursor synthesis. The ratio of the sum of ribosomal protein transcripts to the total number of transcripts ranged from 0.067 to 0.436 and is statistically different between B2 and AD cluster strains (Fig. 2). Expression of translational machinery genes (ribosomal proteins, tRNA, RNA modifying enzymes, etc.) is controlled by the alarmones guanosine tetra- and penta-phosphate (collectively refered to as (p)ppGpp) which are synthesized in response to several stresses (32). (p)ppGpp is synthesized by RelA, binds to DksA, and is degraded by SpoT. Both (p)ppGpp and the (p)ppGpp-DksA complex destabilize weak open promoter complexes such as those for genes of many ribosomal proteins. B2 cluster strains had more *spoT* transcripts, fewer *dksA* transcripts, and about the same number of *relA* transcripts (Fig. 2), which is consistent with lower (p)ppGpp and a higher ribosomal content in B2 cluster strains compared to AD cluster strains.

**Fig 3:**
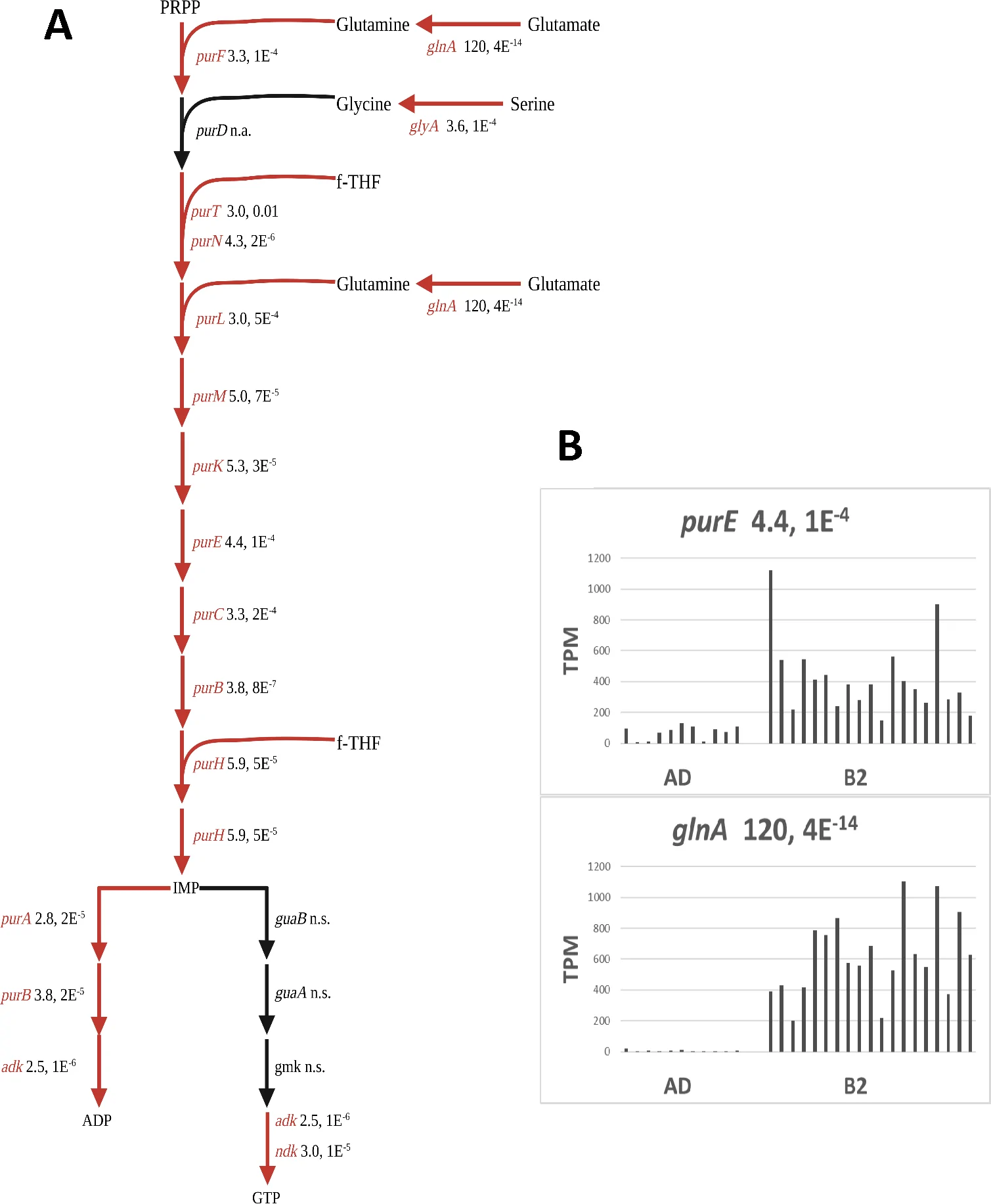
Comparison of transcripts from genes for enzymes that synthesize nucleotides. The first number next to a gene designation is the average number of normalized transcripts from B2 cluster strains divided by the average number of normalized transcripts from AD cluster strains. The second number is the FDR which was determined using the *EdgeR* package. The designation “n.s.” means not significant, i.e., FDR <0.05. The designation “n.a.” means not available: the gene did not appear as a conserved gene. Such genes could be essential but may have two distinct alleles that are scored as different genes.(**A**) Genes for proteins that synthesize purines are shown. The red arrow and red gene designation indicate a B2/AD ratio >1 (usually much greater) and FDR <0.05. The black arrow and gene designation indicate a ratio with an FDR >0.05 or that the information was not available because the gene was not considered conserved. f-THF is *N* ^10^-formyltetrahydrofolate (**B**) Normalized transcript levels for *glnA* and *purE* are shown from 11 AD cluster strains (the lab strain W3110 was excluded) and all 19 B2 cluster strains. In each histogram, values for individual AD and B2 cluster strains are on the left and right, respectively.

Ribosome synthesis is dependent on metabolic pathways that generate nucleotides which require purines, pyrimidines, glutamine, glycine, and *N* ^5^-*N* ^10^-methylene tetrahydrofolate. All but one gene for adenine nucleotide synthesis were more highly expressed in B2 cluster strains (Fig. 3A and B). Curiously, genes for synthesis of guanine nucleotides (Fig. 3A) and pyrimidine nucleotides are not differentially expressed (Table S1). The ranges for *purE* and *glnA* normalized transcripts from individual strains do not overlap between the transcriptomic clusters and is particularly striking for *glnA* (Fig. 3B). The parallel expression of ribosomal proteins and anabolic pathways links translation and metabolism. In summary, B2 cluster strains have higher transcripts for components of the translational machinery.

#### DNA replication and structure

**Fig 4:**
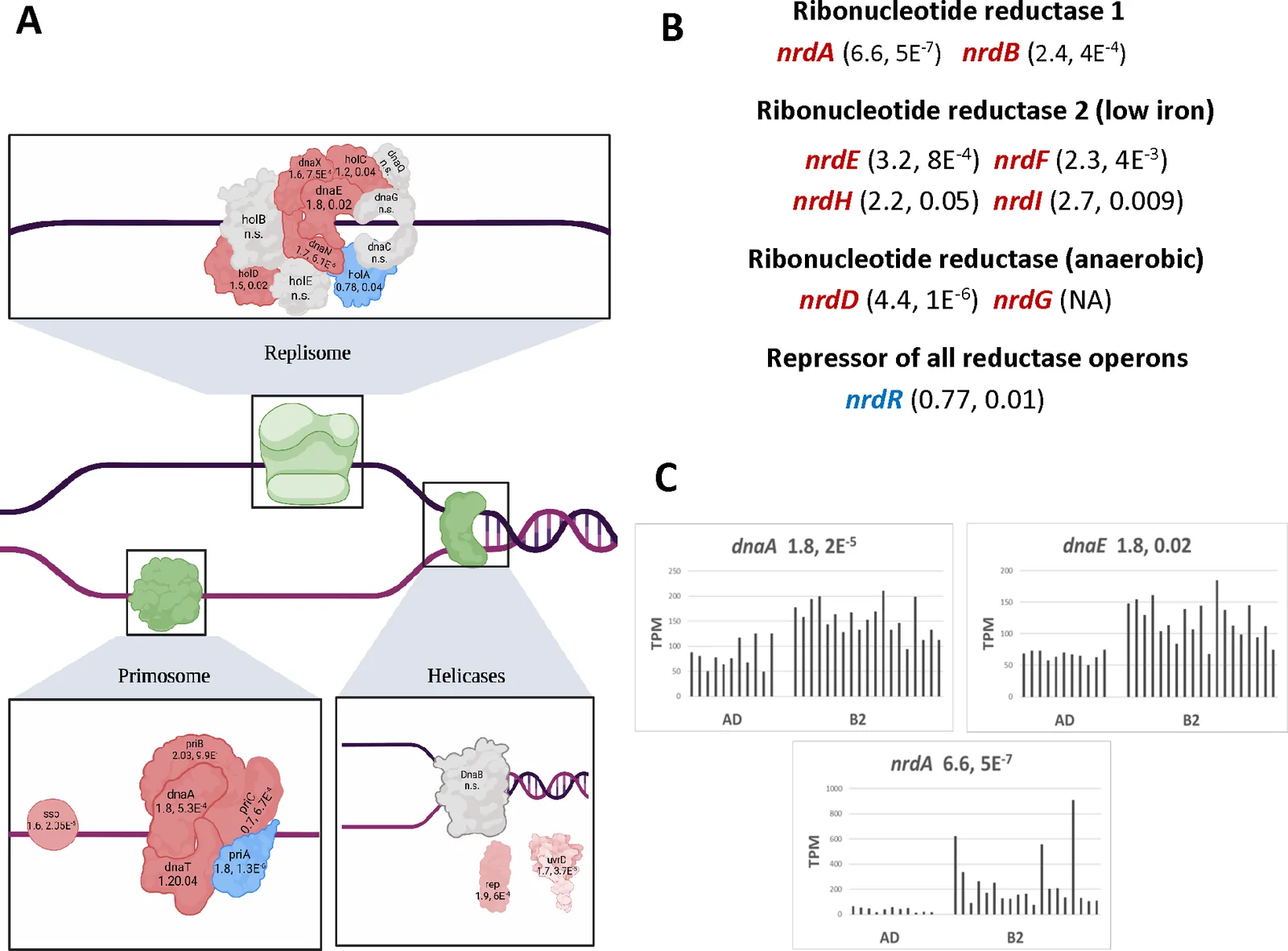
Comparison of transcripts from DNA replication genes. Genes more highly expressed in B2 and AD cluster strains are shown diagrammatically in red and blue, respectively. See the Fig. 3 legend for an explanation of the numbers and abbreviations. (**A**) Expression of genes whose products are part of the primosome and replisome is shown. The structures in the diagram are not meant to be structurally accurate. (**B**) The differentially expressed genes for the three ribonucleotide reductases are all higher in the B2 strains. Expression of the repressor, NrdR, is lower in B2 strains. (**C**) The normalized transcript levels for *dnaA*, *dnaE*, and *nrdA* are shown from individual AD and B2 cluster strains.

The DEGs for translational machinery suggest that B2 strains have a greater capacity for rapid growth, which implies that DEGs for other macromolecular processes should also be affected. Consistent with this prediction, the B2 strains had more transcripts for many, but not all, genes of the core DNA replisome and deoxyribonucleotide synthesis (Fig. 4A through C). Replication requires resolution of replication blocks, such as those caused by transcription complexes, which are resolved by the PriC, Rep, and UvrD helicases (33). B2 cluster strains had higher levels of *rep* and *uvrD* transcripts, and AD cluster strains had more *priC* transcripts (Fig. 4A). These results suggest that B2 and AD cluster strains either use different mechanisms to overcome replication blocks or encounter different types of replication blocks.

**Fig 5:**
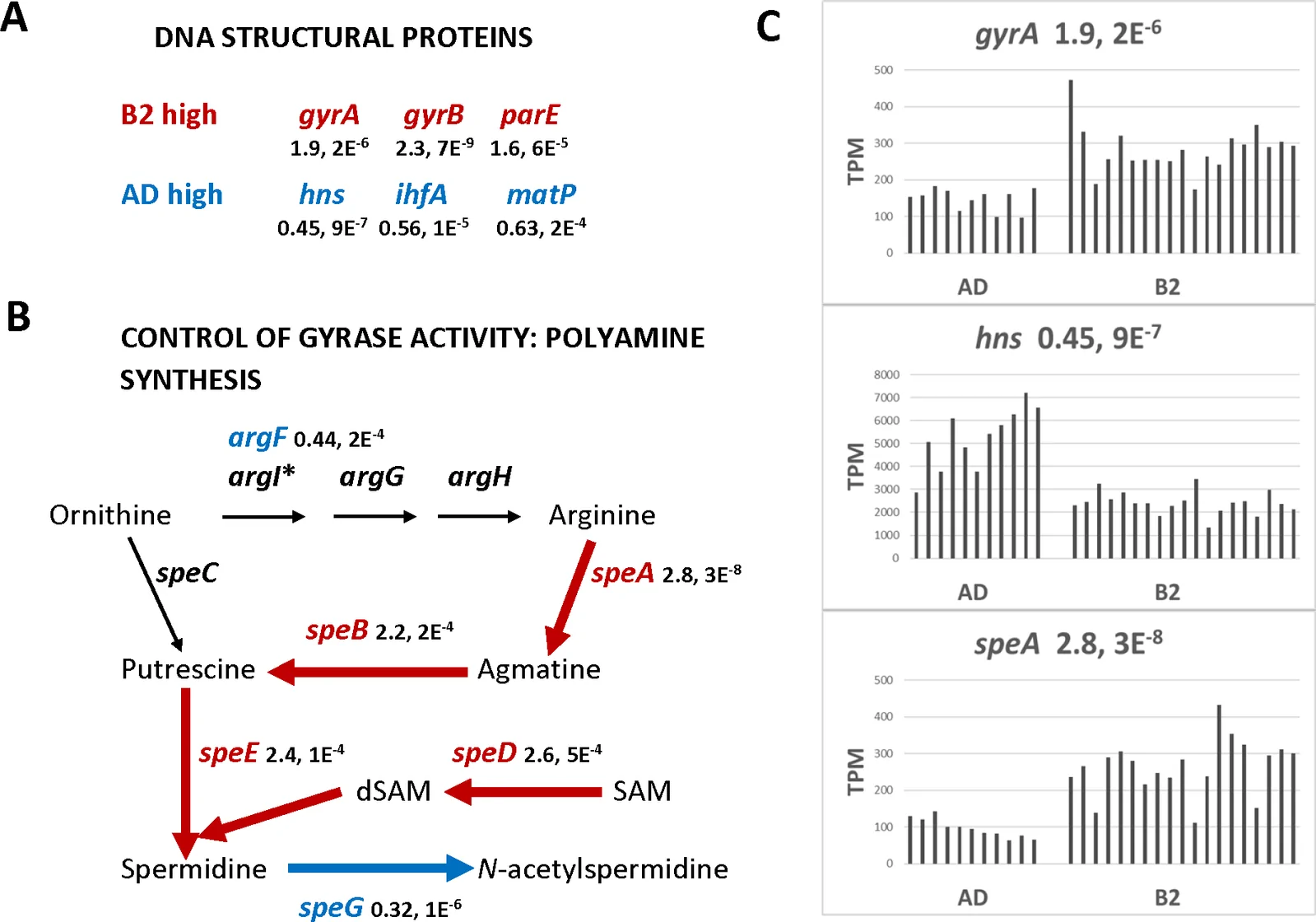
Transcripts for genes of DNA structure. Genes more highly expressed in B2 and AD cluster strains are shown diagrammatically in red and blue, respectively. See the Fig. 3 legend for an explanation of the numbers and abbreviations. (**A**) Genes for nucleoid-associated proteins and proteins that affect supercoiling are shown if the differential expression had FDR <0.001. The differentially expressed genes with FDRs between 0.05 and 0.001 that were higher in B2 cluster strains were *mukB* and *mukE*, and those higher in AD cluster strains were *dps*, *hfq*, and *hupB* (Table S1, DEG.B2vsAD tab). Several genes that affect DNA structure were not differentially expressed, i.e., FDR >0.05: *fis*, *hupA*, *ihfB*, *mukF*, *parC*, *topB*, and *sbmC* (Table S1, DEG.B2vsAD tab). (**B**) The pathway and genes for spermidine synthesis which stimulates DNA gyrase activity. The net effect is to increase putrescine and spermidine in B2 cluster strains. *argI* is a B2-specific gene (39). (**C**) The normalized transcript levels for *gyrA*, *hns*, and *speA* are shown from individual AD and B2 cluster strains.

Supercoiling and nucleoid-binding proteins control DNA condensation (34). B2 strains had more transcripts for the gyrase genes (*gyrA* and *gyrB*) whose products introduce negative supercoiling and fewer transcripts for many genes of nucleoid-associated proteins (*hns*, *ihfA*, *matP*, *dps*, and *hfq*) (Fig. 5A and C, results for only the first three genes are shown). The low molecular weight polyamines—putrescine and spermidine—stabilize negatively supercoiled DNA structure and stimulate gyrase activity (35, 36). B2 strains had more transcripts for the polyamine synthetic genes *speA*, *speB*, *speC*, and *speD*, and fewer transcripts for *speG* whose product modifies spermidine (Fig. 5B and C). Furthermore, phylogenetic group B2 strains lack genes of the two *puu* operons whose products degrade putrescine (37, 38). The normalized transcripts from AD and B2 cluster strains for *gyrA*, *hns*, and *speA* show little overlap for the expression ranges even when the expression ratio is only about twofold (Fig. 5C). The net result of these differences is to increase putrescine and spermidine synthesis and prevent their removal by SpeG and putrescine catabolism. In summary, these results suggest that DNA condensation in B2 cluster strains is more reliant on supercoiling—gyrase expression and increased polyamines which stimulate gyrase activity—whereas DNA condensation in AD cluster strains is more reliant on nucleoid-associated proteins.

#### Transcription

**Fig 6:**
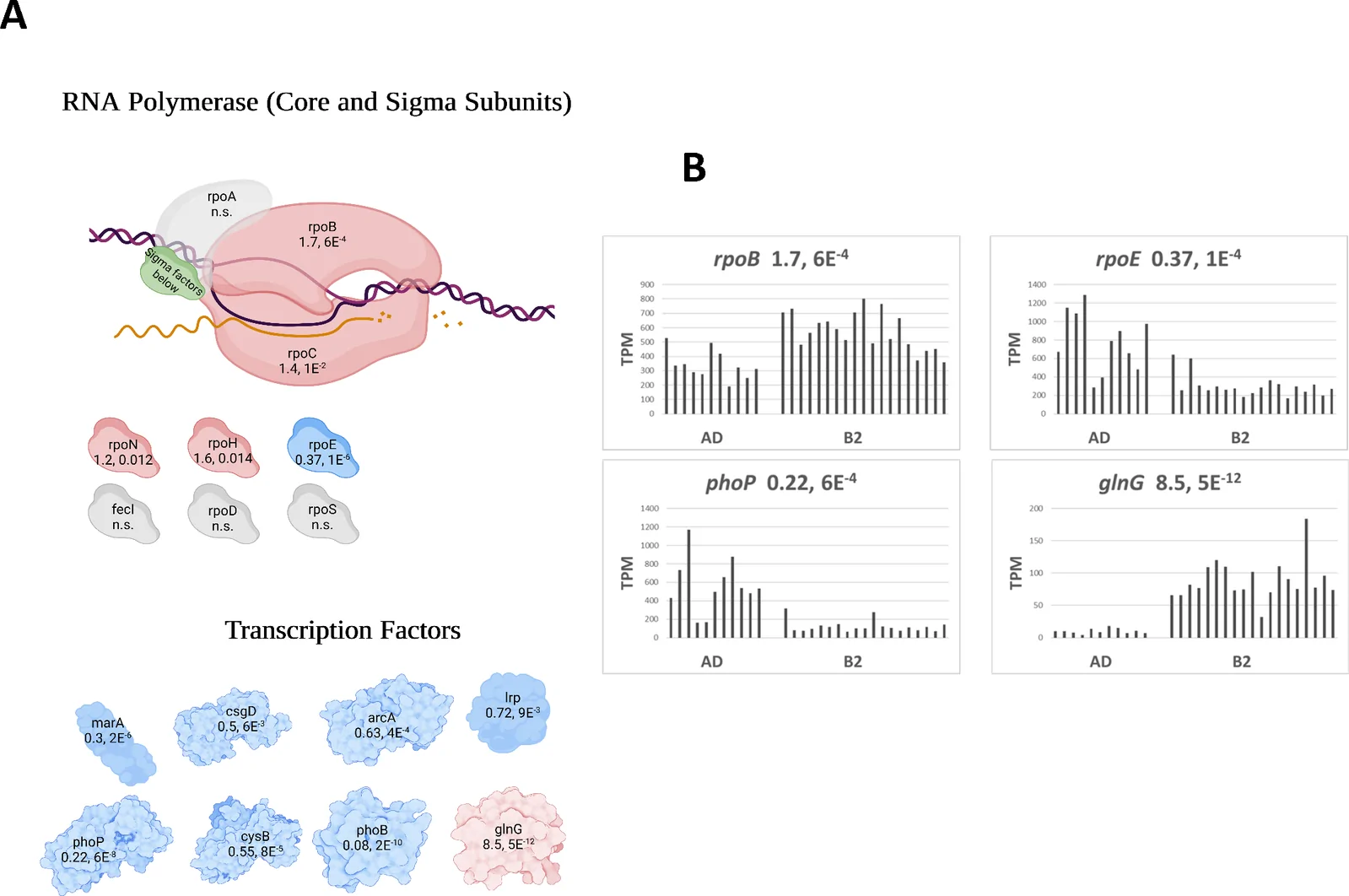
Transcripts for genes of the transcriptional machinery. See the Fig. 3 legend for an explanation of the numbers and abbreviations. (**A**) Expression of genes for core RNA polymerase, the sigma subunits, and stress-responsive transcription factors that are differentially expressed is shown. Genes more highly expressed in B2 and AD cluster strains are shown diagrammatically in red and blue, respectively. The diagrams are not meant to be structurally accurate. (**B**) The normalized transcript levels for *rpoB*, *rpoE*, *phoP*, and *glnG* are shown from individual AD and B2 cluster strains.

Three major factors that control transcription—core RNA polymerase, the promoter-recognizing σ factors, and the DNA-binding TFs—were differentially expressed between B2 and AD cluster strains. B2 strains had more transcripts for two core RNA polymerase genes (*rpoB* and *rpoC*) and two σ subunits genes (*rpoH* and *rpoN*) (Fig. 6A and B). The AD cluster strains had more transcripts for *rpoE* whose product responds to envelope stress (40) (Fig. 6A and B). Seventy-four TFs genes were differentially expressed between the AD and B2 cluster strains (FDR <0.05) (see Table S1, Transcription factor tab for expression of all TF genes and Fig. S1 for a volcano plot of these genes). The products of 12 of these TF genes control more than 10 operons and >13% of regulon genes were differentially expressed in the growth conditions used. Ten of these 12 TF genes were more highly expressed in AD cluster strains (Table 1). The normalized transcripts from AD and B2 cluster strains for *rpoB* and *rpoE* showed some but not extensive overlap, whereas the normalized transcripts for *phoP* and *glnG* showed little or no overlap (Fig. 6B).

**TABLE 1:** Summary of differentially expressed transcription factor genes^*a*^

Genes	Log_2_FC	Log_2_CPM	*P*-value	FDR	Regulon size	Regulon %
*glnG*	−3.08473	8.003008	5.80E-15	5.16E-12	15	41%
*phoB*	3.588802	7.522517	6.74E-13	1.96E-10	32	53%
*phoP*	2.183875	8.59083	1.36E-09	6.02E-08	36	32%
*rpoE*	1.419381	9.480466	5.61E-08	1.13E-06	75	18%
*marA*	1.757421	6.084431	1.01E-07	1.71E-06	32	30%
*cysB*	0.854713	7.764627	1.23E-05	8.38E-05	18	72%
*nac*	−1.85724	5.072371	6.41E-05	0.000333	134	13.3%
*arcA*	0.566224	9.862044	8.80E-05	0.000432	83	22%
*metJ*	0.547261	6.880234	0.001195	0.004014	10	13.3%
*csgD*	1.009655	2.039974	0.002189	0.006516	13	16.7%
*lrp*	0.461569	8.974365	0.003317	0.009233	44	21%
*rpoN*	−0.31829	9.852378	0.004851	0.012809	53	33.3%

^*a*^ Genes presented in this table control more than 10 operons and >13% of the regulon is expressed. All differentially expressed transcription factor genes between B2 and AD cluster strains are presented in Table S1.

In addition to differentially expressed TF genes, we could deduce increased activity in B2 strains of the CpxAR two-component regulatory system that has been implicated in uropathogenesis and responds to envelope stress (41, 42). CpxAR-regulated *cpxP* was more highly expressed in B2 cluster strains (B2 to AD ratio of 8 with an FDR = 0.002), although *cpxA* and *cpxR* were not differentially expressed (Table S1, DEG tab). The Cpx system counters the TFs that are more highly expressed in AD cluster strains: CpxA physically interacts with ArcA—one of the major transcriptional regulators that are more highly expressed in AD strains (Table 1)—and CpxR represses *rpoE* expression (40).

#### Peptidoglycan synthesis and structure

**Fig 7:**
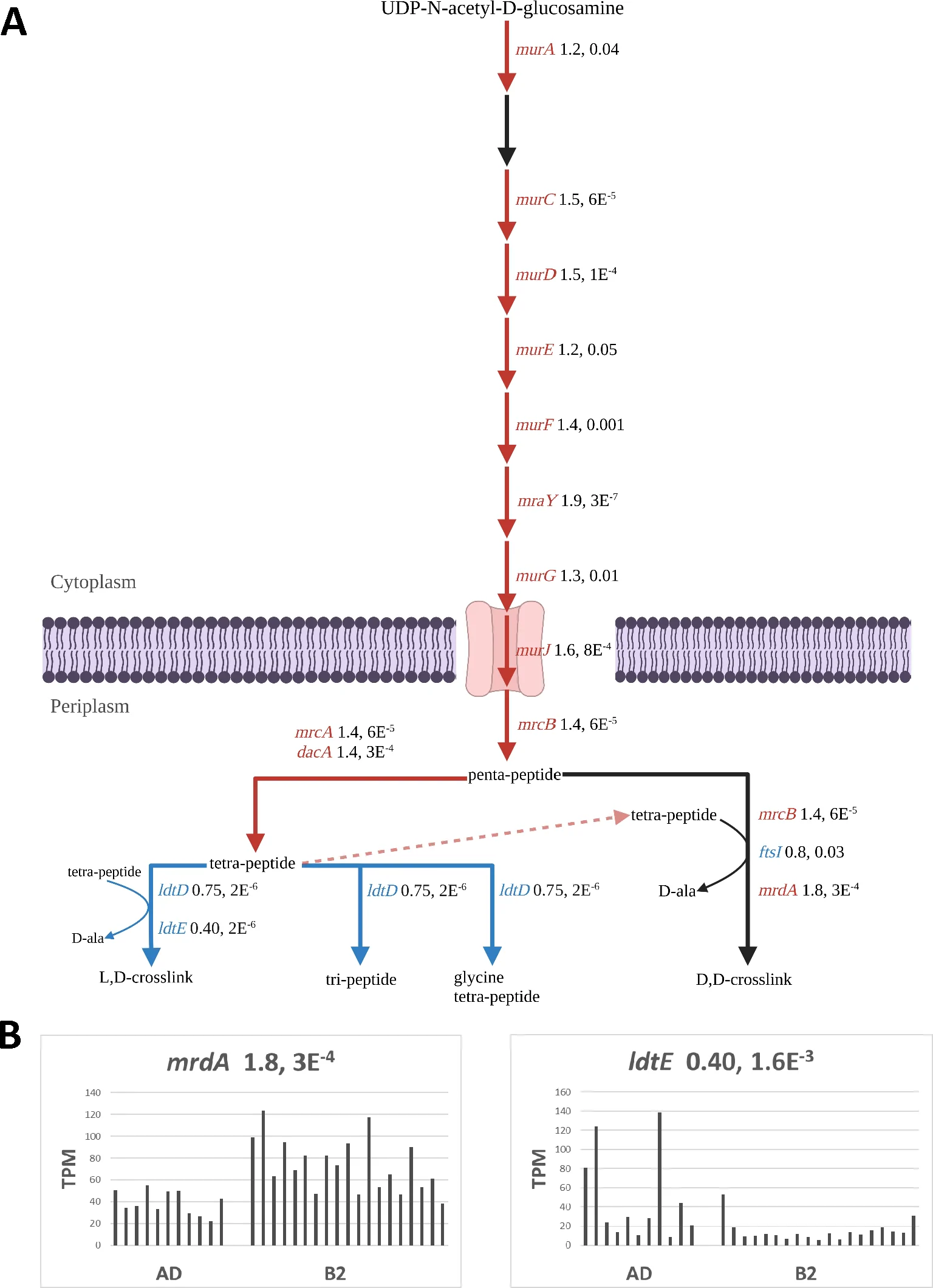
Transcripts for genes of peptidoglycan synthesis. (**A**) Expression of genes for peptidoglycan synthesis is shown starting from UDP-N-acetyl-D-glucosamine. Genes more highly expressed in B2 and AD cluster strains are shown diagrammatically in red and blue, respectively. See the Fig. 3 legend for an explanation of the numbers and abbreviations. Only genes with expression differences with an FDR <0.05 are shown. (**B**) The normalized transcript levels for *mrdA* and *ldtE* are shown from individual AD and B2 cluster strains.

The peptidoglycan layer consists of long-chained polysaccharides crosslinked with short peptides which, when initially synthesized, contain L-alanine, D-glutamate, *meso*-diaminopimelate (an intermediate in lysine synthesis), and two molecules of D-alanine. The three phases of peptidoglycan synthesis are precursor formation, synthesis, and maturation [summarized in reference (40)]. All but one gene of the synthetic phase were statistically more highly expressed in B2 strains, even though the B2 to AD transcript ratios were only 20%–90% higher (Fig. 7A and B). Maturation involves processing of the peptide linkage: one or both D-alanine residues may be removed, and glycine can replace one D-alanine. The maturation genes *dacA*, *mrcA*, *mrcB*, and *mrdA* were more highly expressed in B2 strains, while *ftsI*, *ldtD*, and *ldtE* were more highly expressed in AD strains (Fig. 7A and B). The *ldtD* and *ldtE* genes are associated with stress (43). These results suggest possible differences in peptidoglycan structure between the transcriptomic groups. Such differences could partially explain the difficulty in viral-mediated transduction into group B2 UPEC strains (44, 45).

### Energy metabolism and iron acquisition

#### Energy metabolism

**Fig 8:**
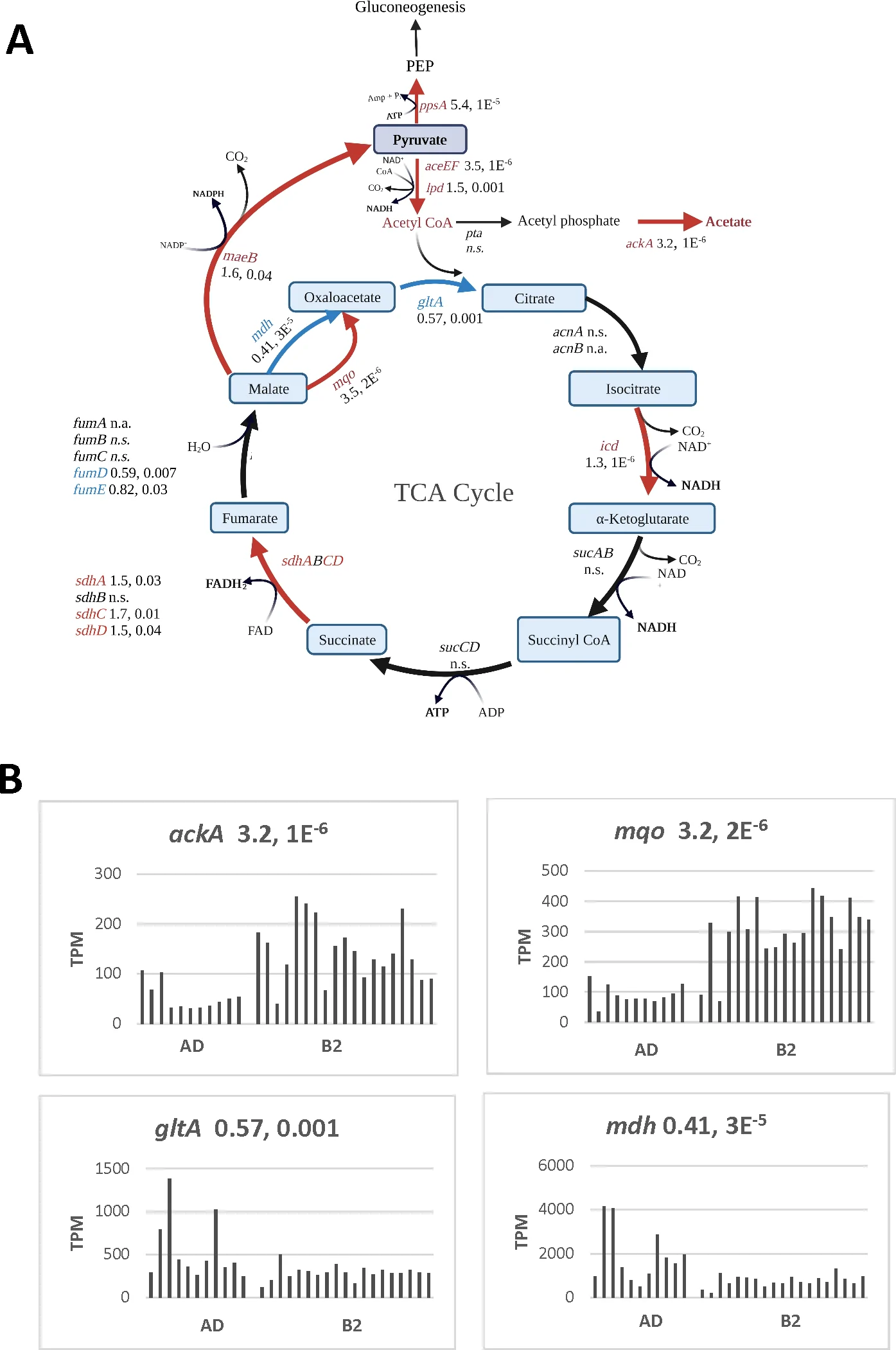
Transcripts from genes for TCA cycle enzymes. (**A**) TCA cycle genes that are more highly expressed in B2 and AD cluster strains are shown diagrammatically in red and blue, respectively. The black designations are for genes that are not differentially expressed between AD and B2 cluster strains. See the Fig. 3 legend for an explanation of the numbers and abbreviations. (**B**) The normalized transcript levels from individual AD and B2 cluster strains are shown for *ackA* and *mqo* which are more highly expressed in B2 cluster strains, and for *gltA* and *mdh* which are more highly expressed in AD cluster strains.

B2 cluster strains had more transcripts for several genes of the tricarboxylic acid (TCA) cycle, electron transport (for NADH dehydrogenase I and II, ubiquinone, menaquinone, and cytochrome *bo_3_* and *bd* oxidase), ATP synthase (for the *atp* operon), acetate formation (*aceEF-lpdA* and *ackA*), and several gluconeogenic genes (*maeB*, *ppsA*, *fbp*, and *glpX*) (Fig. 8; Table S1). The genes that metabolize malate distinguish B2 from AD cluster strains (Fig. 8A and B): in B2 strains, genes that convert malate to pyruvate and of gluconeogenesis are more highly expressed, while in AD strains, genes that complete the TCA cycle are more highly expressed (Fig. 8A and B). This pattern of metabolism is consistent with a previous genetic analysis of energy metabolism in the UPEC strain CFT073: a reliance on amino acid degradation via the TCA cycle and gluconeogenesis instead of glycolysis (46). These results are remarkable because the glucose in the media should inhibit cyclic-AMP synthesis which is required for expression of most TCA cycle enzymes, the cytochrome *bo_3_* oxidase specified by the *cyoABCD* operon, the *aceEF-lpdA* operon, and *glpX* [summarized in reference (40)]. This apparent paradox is explained by a flux balance analysis of growth in a complex medium with glucose and amino acids that showed that the fastest growth is achieved with preferential use of amino acids which reduces glucose uptake (rapid amino acid degradation increases intracellular pyruvate which, in turn, inhibits glucose transport) and increases acetate overflow (47). The reduced glucose uptake could account for expression of genes that require cyclic-AMP. For glycolytic genes in either the Embden-Meyerhof-Parnas or the oxidative arm of the pentose-phosphate pathway, group B2 strains had more transcripts for *ptsG*, *gapA*, *tpi*, and *gndA* and fewer transcripts for *gpmM*, *eno*, *pykA*, and *zwf*. These DEGs do not suggest an obvious pattern. Despite these results, evidence exists that carbohydrate catabolism is important for UTI89 under certain circumstances, which suggests that our understanding of carbohydrate catabolism in UPEC strains is incomplete (48, 49). In summary, B2 cluster strains appears to have a pattern for the genes of energy metabolism that differs from that of AD cluster strains.

#### Iron acquisition

**Fig 9:**
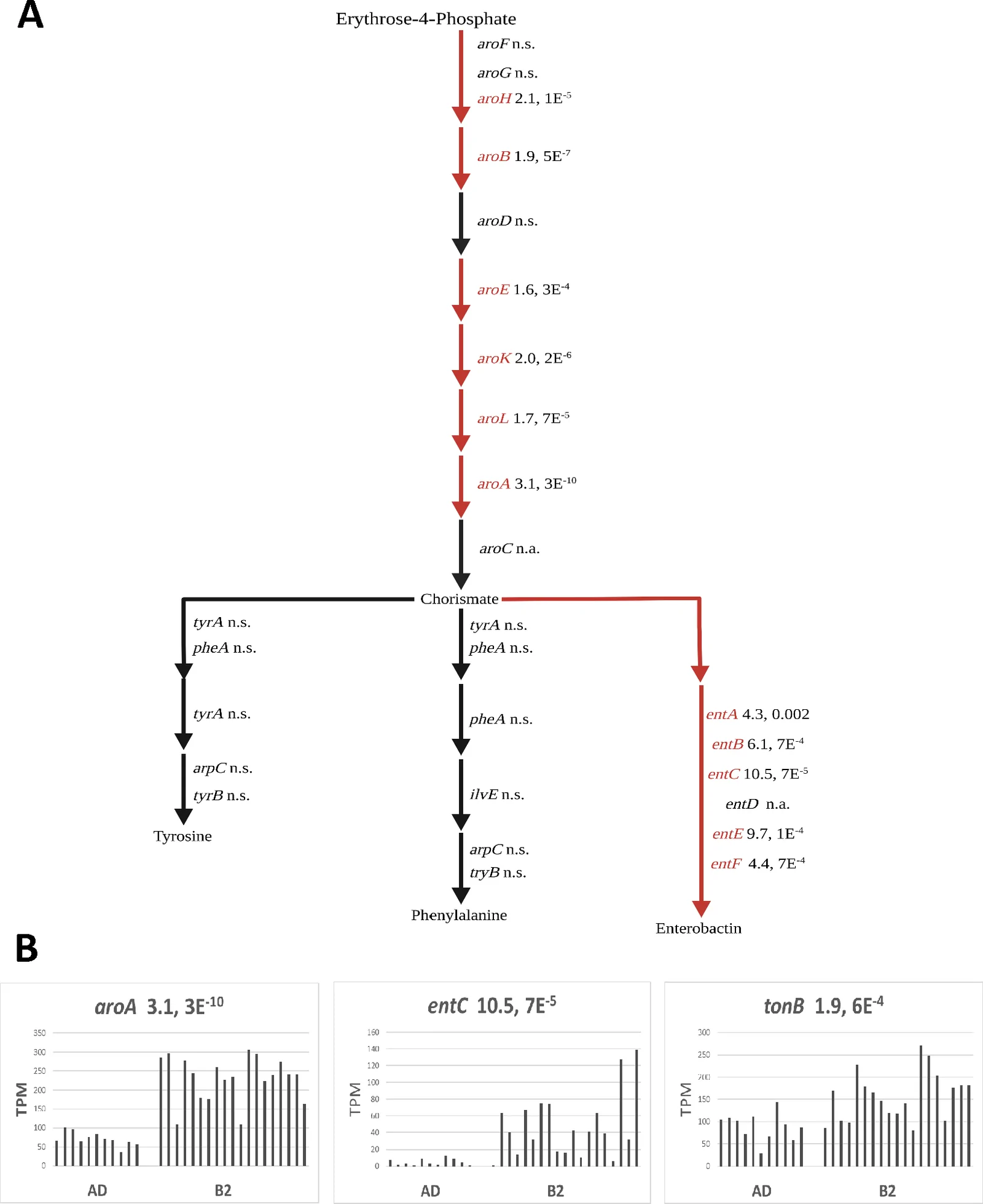
Transcripts for iron acquisition genes. (**A**) Expression of genes for synthesis of enterochelin and two other compounds, tyrosine and phenylalanine, derived from chorismate is shown. The red designations indicate genes that are more highly expressed in B2 cluster strains, and the black designations are for genes that are not differentially expressed between AD and B2 cluster strains. See the Fig. 3 legend for an explanation of the numbers and abbreviations. (**B**) The normalized transcript levels for *aroA*, *entC*, and *tonB* are shown from individual AD and B2 cluster strains. All are more highly expressed in B2 cluster strains.

Iron is important for several components of energy-generating pathways and, not surprisingly, iron acquisition is a crucial virulence factor (50). B2 cluster strains had more transcripts for most of the core iron acquisition genes: (i) energy-transducing iron-chelate transport (*tonB*, *exbB*, and *exbD*), (ii) transport systems for ferric citrate (*fec* genes), ferrous iron (*feo* genes), ferric enterochelin transport (*fep* genes), and ferrichrome (*fhu* genes), and (iii) synthesis of enterochelin (*ent* genes) and enterochelin precursors (several *aro* genes) (Fig. 9A and B; Table S1, iron acquisition tab). Fur complexed to ferrous iron represses many of these genes, but *fur* was not differentially expressed between B2 and AD cluster strains. Therefore, greater expression of iron acquisition in B2 cluster strains implies lower intracellular iron. In summary, B2 cluster strains had higher expression of numerous iron acquisition genes.

## DISCUSSION

### The relation between phylogenetics and transcriptomics

Our first major conclusion is that *E. coli* phylogenetic group B2 strains, which are associated with several pathologies, had a distinct core gene transcription pattern when grown in a nutrient-rich environment. Almost half of the core genes were differentially expressed between B2 and AD cluster strains. The transcriptomic pattern generally followed phylogeny, which is based on the presence of specific chromosomal genes, although an occasional group A strain possessed the B2 transcriptome pattern, and vice versa (Fig. 1A). The malleability and discreteness of the transcriptome patterns suggest that the transition involves only a few genes, but our results do not suggest which genes are responsible for the transcriptional rewiring. One study in particular—the longest-running microbial evolution experiment—described a transcriptomic rewiring that affected some of the same genes that distinguish B2 and AD cluster strains: genes that control DNA supercoiling and the stringent response (51). The evolved changes were the result of daily cycles of starvation and growth which is likely to be relevant for pathogens that must survive and replicate in different, constantly changing, and nutritionally challenging environments. Recent results suggest that (p)ppGpp controls *gyrA* expression, which provides a mechanistic link between DNA supercoiling and the stringent response (52). A possible explanation for the distinct B2-type transcriptome is lower (p)ppGpp levels and more DNA supercoiling. Additional experiments are required to determine whether (p)ppGpp and supercoiling are sufficient to account for the distinct B2 transcriptome.

Evolutionary analysis has suggested that group B2 strains diverged early from other *E. coli* strains and have lost several stress-adaptive genes, which implied a different adaptive trajectory (21). In addition to loss of stress-adaptive genes, our results show that B2 strains have lower expression of stress-adaptive core genes in a relatively stress-free nutrient-rich environment (Table 1). Despite their lower expression in B2 cluster strains, several of these stress-responsive TFs are important during virulence of *E. coli* and other pathogens. The *phoP* gene is important for virulence of the uropathogen CFT073 and *Salmonella* and has been associated with control of the proton motive force, intracellular survival, and responses to low pH, low magnesium, and antimicrobial peptides (53) (54, 55). The phosphate-sensing PhoR-PhoB system has also been associated with *E. coli* virulence and is required for expression of a gene that regulates type I pili synthesis in CFT073 (56) (57, 58). RpoE has been implicated in virulence of *Salmonella* and *E. coli*, including UPEC strains (59 – 62). RpoE is stress-induced sigma subunit for RNA polymerase that responds to high temperature and osmolarity, misfolded proteins in the outer membrane, periplasm, and inner membrane, metal ion exposure, entry into stationary phase, and elevated (p)ppGpp (40). Finally, the stringent response has been associated with virulence and antibiotic resistance in a variety of pathogens (55, 63 – 68). Further experiments are required to determine whether B2 and AD strains differ in sensing and responding to a variety of stresses.

Little is known about the metabolic and physiological differences between the phylogenetic groups. Our results showed that B2 cluster strains had higher expression for several metabolic genes, including those for macromolecular synthesis, several components of the TCA cycle, electron transport, and acetogenesis. We have recently observed that two group B2 UPEC strains grow better in urine and dilute lab medium than a group A NPEC strain, which implies better nutrient acquisition (29). The higher levels of iron acquisition genes in group B2 strains are consistent with this observation (Fig. 9). Better nutrient acquisition will support faster growth in nutrient-poor environments, which is consistent with the faster growth of UPEC strains in urine compared to NPEC strains (27, 28). We propose that efficient nutrient acquisition and the capacity for faster growth—higher expression of TCA cycle enzymes, several electron transport components, ATP synthase, and iron acquisition in B2 strains—are coupled properties that enhance growth in urine.

### Distinguishing UPEC and NPEC strains

Our second major conclusion is that transcriptomic analysis of core gene expression did not identify major differences between UPEC and NPEC strains within the B2 cluster. We cannot conclude that B2-specific properties are sufficient for virulence because our analysis was not capable of identifying strain-specific adaptations to individual hosts. S. Hultgren and colleagues have emphasized the importance of host-specific properties and host-pathogen interactions which limit generalizations about virulence (22). Such considerations would be expected for an opportunistic pathogen: the virulence mechanism may be dependent on the specific host, and B2 strains may have greater adaptability.

### The problem with model UPEC strains

The vast majority of UPEC studies have focused on the model group B2 strains UTI89 and CFT073 with few studies of strains in other groups. Our analysis showed these two strains were among four UPEC strains that did not cluster within the B2 or AD transcriptome patterns. A distinctive property of CFT073 was 47-fold overexpression of *phoB* compared to other B2 strains. The only other strain with a similar level of *phoB* was W3110. These two strains have been passaged in labs for decades, and the *phoB* hyperexpression in both strains may be a property of laboratory evolution in the low phosphate amino acid-rich broths that are commonly used in labs, i.e., L-broth (LB). UTI89 has also been extensively passaged, which may have altered its transcriptome. The outliers’ transcriptomes may be inherent to these strains when they were originally isolated, and given the diversity of host environments, these strains may provide information on some extreme environments. However, their properties may also result from lab evolution, and some of their properties are not necessarily shared with other UPEC strains.

### Does the group B2-type transcription pattern contribute to virulence?

Group B2 strains are often associated with UTIs and a variety of pathologies. Virulence is necessarily a property of both the bacteria and the host response to the infection, which degrades with age and presumably shows substantial variation between individuals. With respect to the bacteria, we have established that B2 strains possess a unique transcription pattern when grown in a nutrient-rich medium. In addition, B2 strains also possess a unique set of non-core accessory genes that define the B2 group. Both properties of B2 strains are linked if the transcription factors encoded by core genes control expression of the accessory genes. Mobley and colleagues have proposed a distinct transcription pattern for UPEC strains isolated from a physiologically relevant environment (23, 69). Most, but not all, of these strains were group B2, and they argued that the transcription pattern is not a property of B2 strains (69). We can reconcile our results with this conclusion if the non-B2 strains exhibit a B2 transcription pattern, either by mutation or adaptation to a physiologically relevant environment. Nonetheless, several observations suggest that the B2 transcription pattern does contribute to virulence. First, the transcriptome of UPEC strains analyzed from the host shows high levels of the translational machinery (23, 69), which our analysis showed is a property of B2 cluster strains. Second, analysis of the pathway requirements for the model strain CFT073 indicates use of the TCA cycle and gluconeogenesis (46), and the same pattern is suggested by the B2 transcription pattern. Third, acetogenesis is a property of UPEC strains and acetate is a UTI biomarker (28, 70, 71). Acetogenesis is suggested by the B2-specific transcriptome. Fourth, CFT073 has been shown to have high levels of iron acquisition genes when grown in urine (28). Our results suggest that B2 strains have high endogenous levels of iron acquisition genes. The contribution of any single factor, such as a B2-type transcriptome, to virulence cannot be precisely determined. However, because UPEC gene expression in physiologically relevant environments parallels the gene expression pattern of B2 strains that we observed, we propose that the B2 transcription pattern is one, but not the only, factor that contributes to virulence. Further studies will be required to determine where and when during an infection the transcription pattern described in this study is important.

### Limitations of this study

The major limitations of our study are conceptual. Because of the need to grow UPEC and NPEC strains at similar rates, the bacteria were grown in a non-physiological environment. Understanding virulence will require analysis from bacteria grown in “more relevant” environments. Our results did not find differences between UPEC and NPEC strains, but such differences may exist. Our results suggest differences in the expression of stress-responsive transcription factor genes during growth in a nutrient-rich lab medium, but provide no information on differences in the responses to stress between the B2 and AD cluster strains. These topics require further study.

## MATERIALS AND METHODS

### Strains, bacterial growth, and RNA isolation

Strains and their sources are listed in Table 2. Strains were grown to single colonies overnight on LB. A culture was inoculated from a single colony into 10 mL of GT media (0.5% glucose, 1% tryptone, 0.25% NaCl) until mid-log growth phase, collected by a table top centrifugation at maximum speed for 10 min at 4°C, and the cell pellets were frozen at −80°C.

**TABLE 2:** Strain list

Strain	Group	NPEC/UPEC	Source
ECOR 4	A	NPEC	(72)
ECOR 6	A	NPEC	(72)
ECOR 8	A	NPEC	(72)
ECOR 11	A	UPEC	(72)
ECOR 14	A	UPEC	(72)
ECOR 16	A	NPEC	(72)
ECOR 23	A	NPEC	(72)
ECOR 51	B2	NPEC	(72)
ECOR 52	B2	NPEC	(72)
ECOR 53	B2	NPEC	(72)
ECOR 54	B2	NPEC	(72)
ECOR 55	B2	UPEC	(72)
ECOR 56	B2	UPEC	(72)
ECOR 60	B2	UPEC	(72)
ECOR 61	B2	NPEC	(72)
ECOR 62	B2	UPEC	(72)
ECOR 64	B2	UPEC	(72)
ECOR 66	B2	NPEC	(72)
ECOR 44	D	NPEC	(72)
ECOR 47	D	NPEC	(72)
ECOR 48	D	UPEC	(72)
ECOR 49	D	NPEC	(72)
ECOR 50	D	UPEC	(72)
CFT073	B2	UPEC	(73)
KE4	B2	UPEC	(74)
KE21	B2	UPEC	(74)
KE24	B2	UPEC	(74)
KE25	B2	UPEC	(74)
KE26	B2	UPEC	(74)
LRPF007	B2	UPEC	(74)
LRPF62	B2	UPEC	(74)
PNK004	D	UPEC	(75)
PNK006	A	UPEC	(75)
UTI89	B2	UPEC	(76)
W3110	A	NPEC	Lab strain

Frozen cell pellets were thawed on ice, suspended in 0.7 mL of buffer RLT (from Qiagen RNeasy Mini kit), and mechanically lysed using a bead beater (FastPrep-24 Classic from MP Biomedical) set at the highest setting for three 45-s cycles with a 5-min rest period on ice between cycles. Once lysed, a Qiagen RNeasy Mini Kit was used to collect RNA and treated with RNase-free DNase to remove DNA. Samples had to have at least 10 µg of RNA as determined by A260 (nanodrop). To remove the DNase and concentrate the RNA, a GeneJet RNA Isolation and Concentration Kit was used. We continued only if purified RNA was ≥1 µg/µL (quantified by A_260_ by nanodrop) and the A_260_/A_230_ ratio was between 1.9 and 2.2. Then, RNA was quality checked using a Qubit 4 fluorometer (ThermoFisher) and we continued only with an RNA IQ score ≥8.0. To ensure the RNA was not degraded, 1 µg was added to a 1.0% agarose gel and electrophoresed for 1 h at 90V. If the rRNA bands were clear with no smearing, then 1 µg RNA was subjected to reverse transcription. cDNA was used as a template for qPCR using *arcA* as a target and *rpoD* as a library control. RNA was submitted if the no reverse transcriptase control (used to test for small amounts of contaminating DNA) returned at greater than 30 cycles, and the cDNA-containing reactions produced detectable amplification at ≤20 cycles. When the RNA passed all of the tests above, it was submitted to the genomics core facility at the University of Texas at Dallas for RNA sequencing. The core performed rRNA removal (RiboMinus Transcriptome Isolation Kit or RiboCop bacterial rRNA depletion—Lexogen), library preparation (Stranded Total RNA Prep—Illumina), and single-end Illumina sequencing.

### Analysis of the RNA sequences

We compared the within-strain variances for five strains—CFT073, PNK004, PNK006, UTI89, and W3110—for which RNA was isolated three times to the between-strain variances. The within-strain gene variances were much smaller than the between-strain variances (Fig. S2). Therefore, we isolated RNA from most strains only once. The logic of this analysis was that analyzing more strains was more important than analyzing fewer strains with more replicates. We validated differences for *phoP* with quantitative reverse transcriptase PCR (Fig. S3), and for *glnA* with a *glnA-gfp* fusion as part of a separate study (77).

Annotations for KE and LRPF strains were performed by National Center for Biotechnology Information (NCBI) using pan-genome analysis pipeline (PGAP, version 6.4). The gene annotation files of the other 28 strains were downloaded from FTP server in NCBI (https://ftp.ncbi.nlm.nih.gov/genomes/). All gff3 files were used to generate a pan-transcriptome by *Roary* (version 3.11.2) (78) with default parameter setting to assign all transcripts to 17,098 gene clusters. Among them, 2,689 clusters had ortholog genes in at least 34 strains and were defined as the core gene set. We used *Kallisto* (version 0.45.1) (79) to quantify gene expression from RNA-seq data of each strain by using annotated transcripts from all strains as reference. The gene expression of each cluster was defined as the sum of expression from all transcripts belonging to this cluster. To perform DEG analysis, we used *EdgeR* (version 3.40.2) (80, 81) to analyze read counts of clusters in core gene set across all strains. DEGs were detected with an adjusted *P*-value less than 0.05. *ClusterProfiler* (version 4.6.0) (82, 83) was used to perform function and pathway enrichment analysis. K-mean clustering (*n* = 3) was performed with all core gene-related clusters in *R* for grouping all strains.

## Data Availability

RNA sequencing data is available at GSE235964 through NCBI GEO.

## SUPPLEMENTAL MATERIAL

Figures S1 to S3 spectrum.02085-23-s0001.docx S1: volcano plot of transcription factor genes; S2: Variation between strains versus between replicates; S3: RNAseq validation of phoP by quantitative reverse transcriptase PCR.

Table S1-S3 legends spectrum.02085-23-s0002.docx Overall description of contents of the supplemental Excel files.

Table S1 spectrum.02085-23-s0003.xlsx Differentially expressed genes between AD and B2 cluster genes.

Table S2 spectrum.02085-23-s0004.xlsx Gene expression between pathogens and nonpathogens within group B2 cluster strains.

Table S3 spectrum.02085-23-s0005.xlsx Differentially expressed genes between pathogenic and nonpathogenic strains of AD cluster strains.

ASM does not own the copyrights to Supplemental Material that may be linked to, or accessed through, an article. The authors have granted ASM a non-exclusive, world-wide license to publish the Supplemental Material files. Please contact the corresponding author directly for reuse.
