# Supplementary material for: The distinct transcriptome of virulence-associated phylogenetic group B2 Escherichia coli
Source: Microbiol Spectr. 2023 Sep 19;11(5):e02085-23. doi: 10.1128/spectrum.02085-23 (PMC10580932; doi:10.1128/spectrum.02085-23)
Supplement: Figures S1 to S3 — S1: volcano plot of transcription factor genes; S2: Variation between strains versus between replicates; S3: RNAseq validation of phoP by quantitative reverse transcriptase PCR. [file spectrum.02085-23-s0001.docx]

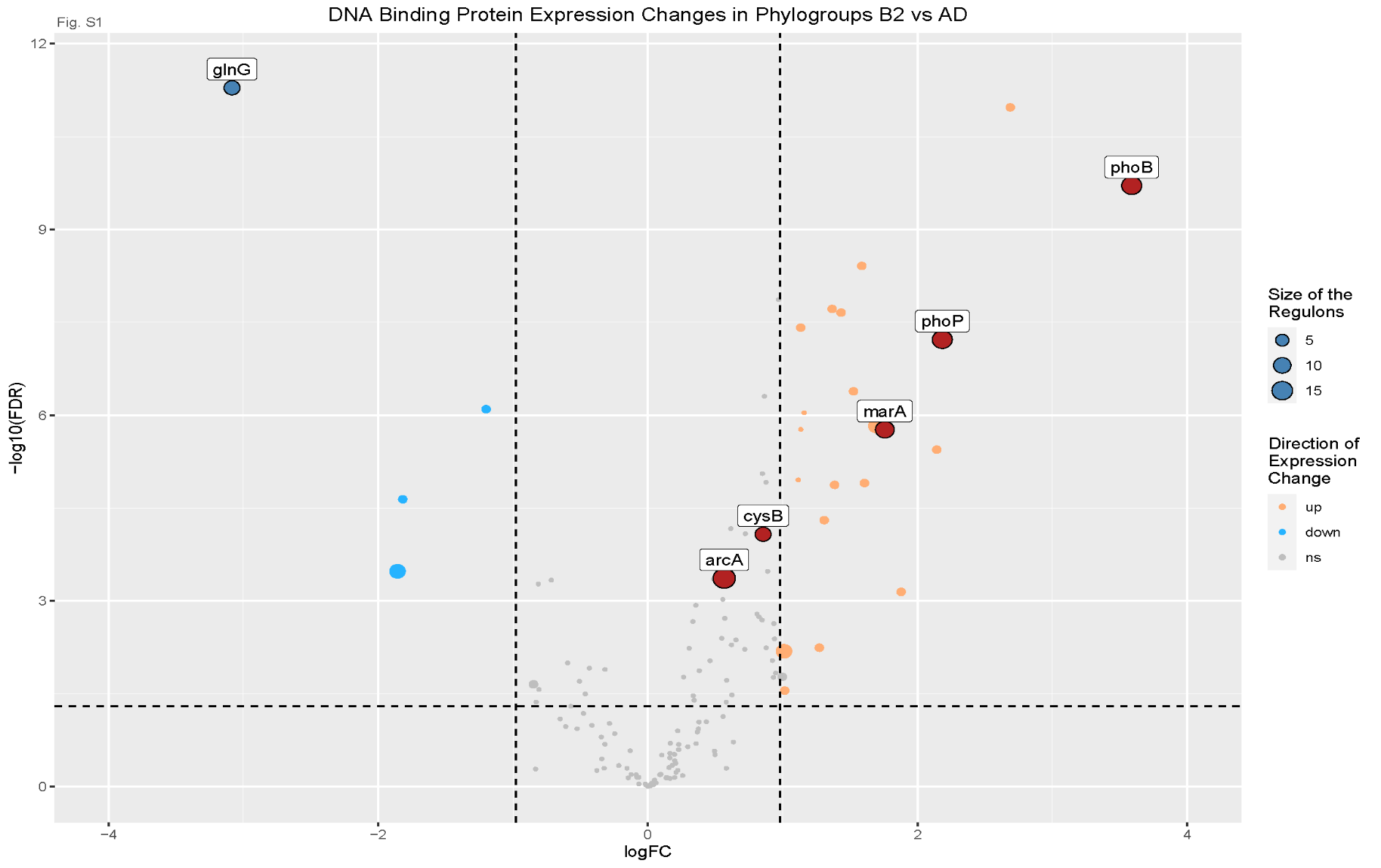


Figure S1. Volcano plot of transcripts for all DNA-binding transcription factors. The vertical dashed lines for x-axis values show the boundaries for 2-fold changes, while above the horizontal dashed line for the y-axis is for FDR < 0.05.


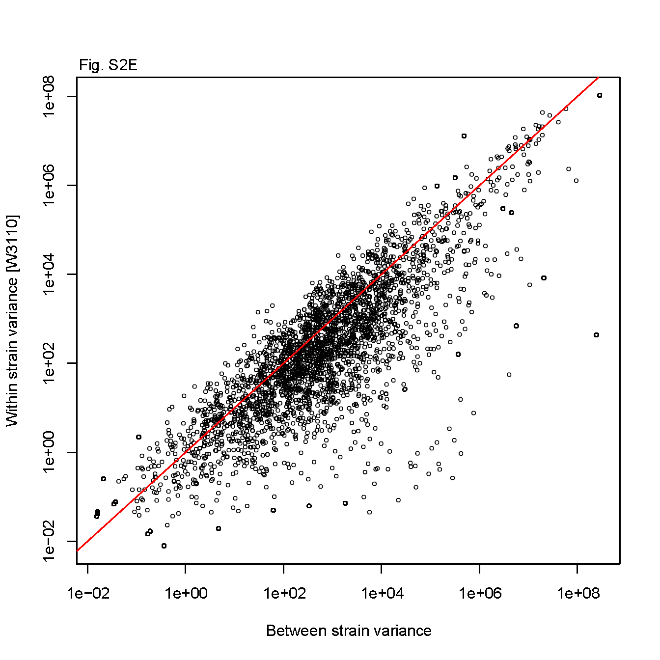

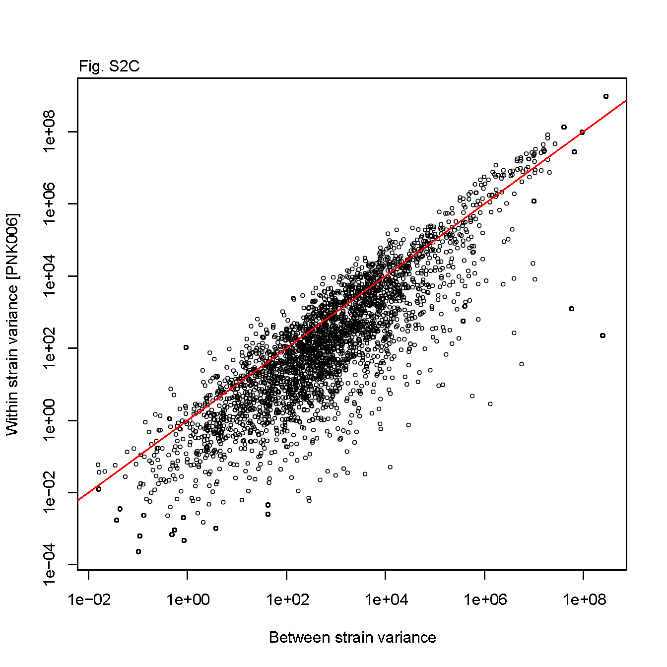

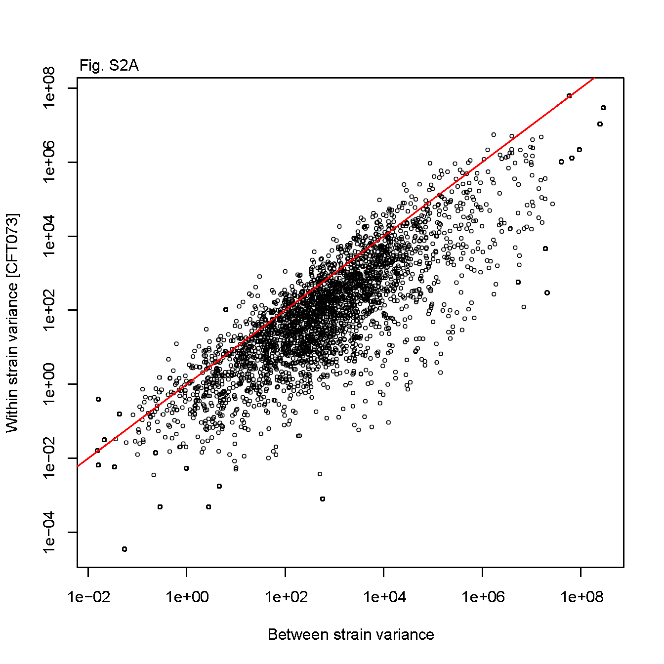

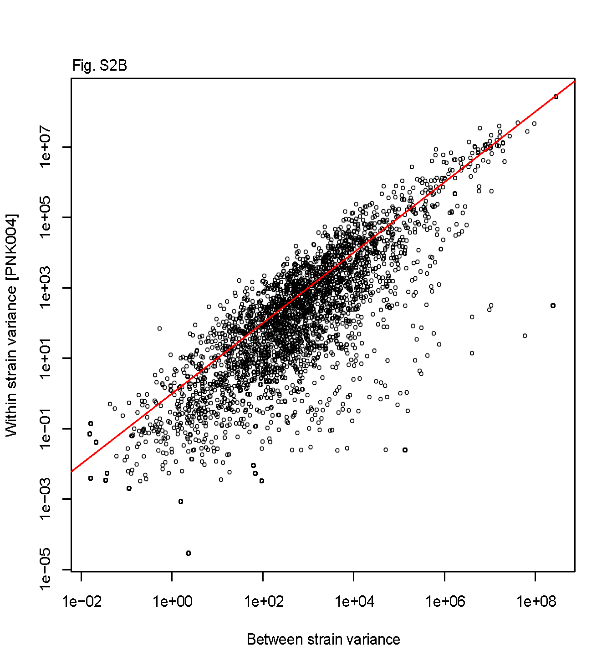

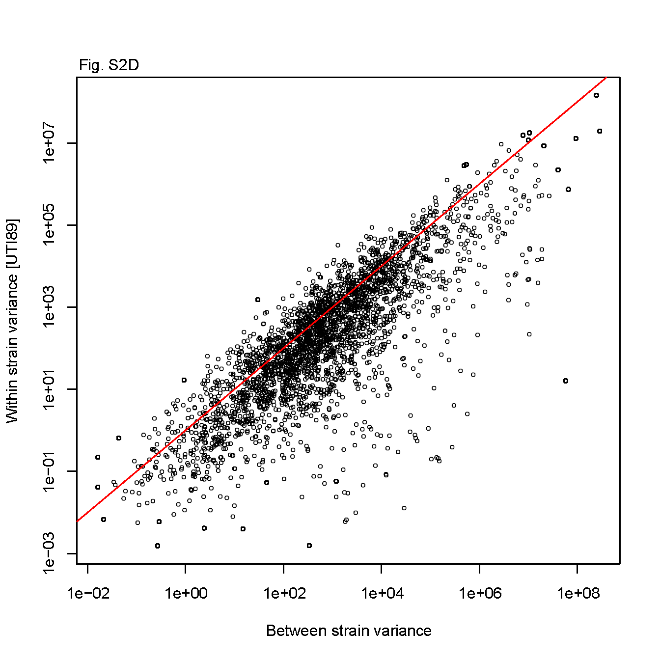


Figure S2. Plots of within strain and between strain variances. (A) CFT073; (B) PNK004; (C) PNK006; (D) UTI89; and (E) W3110. RNA was independently extracted three times from each strain. Because the between strain variances were larger than the within strain variances, RNA was extracted only once from the other 31 strains.


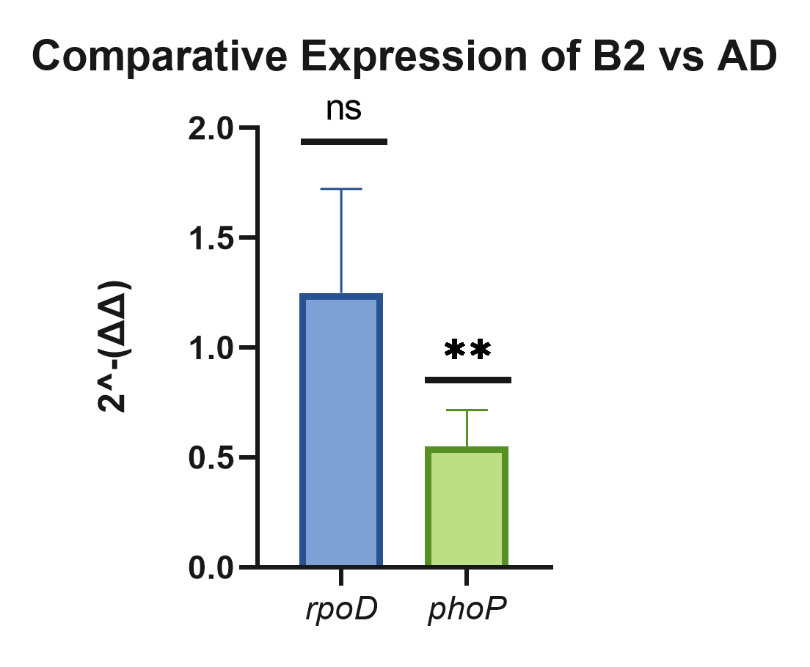


Figure S3. Validation of RNAseq results for *phoP* expression.

RNAseq analysis showed 4-fold higher phoP expression in AD cluster strains. The results of quantitative reverse transcriptase PCR shown here indicates that *phoP* was 2-fold higher in the average of nine AD cluster strains compared to the average in 12 B2 strains. For this experiment, *rpoD* was used as a control. The PCR method is subject to greater variation because the fold change is based on a ratio instead of an absolute number.
