# Supplementary material for: The distinct transcriptome of virulence-associated phylogenetic group B2 Escherichia coli
Source: Microbiol Spectr. 2023 Sep 19;11(5):e02085-23. doi: 10.1128/spectrum.02085-23 (PMC10580932; doi:10.1128/spectrum.02085-23)
Supplement: Table S1-S3 legends — Overall description of contents of the supplemental Excel files. [file spectrum.02085-23-s0002.docx]

**Table S1:** Comparison of genes present in at least 34 of 35 strains (core genes). The DEG.B2vsAD tab is sorted from lowest to highest false discovery rate (FDR) p value.

The iron acquisition tab contains only the genes required in iron chelator synthesis and is sorted based on alphabetical gene names.

The B2vsAD.GSEA.GOMF tab contains the statistically significant (FDR q value <0.05) molecular function gene ontology (GOMF) terms sorted from the lowest to highest false discovery rate q value (qvalues).

The B2vsAD.GSEA.GOBP tab contains the statistically significant (FDR q value <0.05) biological process gene ontology (GOBP) terms sorted from the lowest to highest false discovery rate q value (qvalues).

The B2vsADTranscription factor list tab contains a list of the transcription factors as described by the *Escherichia coli* K-12 transcriptional regulatory database (regulondb.ccg.unam.mx) sorted from lowest to highest false discovery rate (FDR) p value.

**Table S2:** Comparison of the gene expression changes between the B2 pathogens and the non-pathogens excluding any “y genes” with no known function. Data is sorted from lowest to highest false discovery rate (FDR) p value. No gene ontology terms were enriched in either the molecular function or biological process sets.

**Table S3:** Comparison of the gene expression changes between the AD pathogens and the non-pathogens excluding any “y genes” with no known function. No genes were found to be significantly differentially expressed.

The ADUvsADN.GSEA.GOMF tab contains the statistically significant (FDR q value <0.05) molecular function gene ontology (GOMF) terms sorted from the lowest to highest false discovery rate q value (qvalues).

The ADUvsADN.GSEA.GOBP tab contains the statistically significant (FDR q value <0.05) biological process gene ontology (GOBP) terms sorted from the lowest to highest false discovery rate q value (qvalues).
